# Protocol for the Use of a Novel Bioreactor System for Hydrated Mechanical Testing, Strained Sterile Culture, and Force of Contraction Measurement of Tissue Engineered Muscle Constructs

**DOI:** 10.3389/fcell.2021.661036

**Published:** 2021-04-13

**Authors:** Sarah M. Somers, Warren L. Grayson

**Affiliations:** ^1^Translational Tissue Engineering Center, Johns Hopkins University School of Medicine, Baltimore, MD, United States; ^2^Department of Biomedical Engineering, Johns Hopkins University School of Medicine, Baltimore, MD, United States; ^3^Department of Material Sciences and Engineering, Johns Hopkins University School of Engineering, Baltimore, MD, United States; ^4^Department of Chemical and Biomolecular, Johns Hopkins University School of Engineering, Baltimore, MD, United States; ^5^Institute for NanoBioTechnology (INBT), Johns Hopkins University School of Engineering, Baltimore, MD, United States

**Keywords:** bioreactors, skeletal muscle, tissue engineering, mechanobiology, electrospun fibrin

## Abstract

Bioreactor systems are built as controlled environments for biological processes and utilized in the field of tissue engineering to apply mechanical, spatial, and chemical cues to developing tissue grafts. Often the systems are applied to instruct differentiation and maturation of the cells grown inside. Perhaps the most obvious targets for strain and compression-based bioreactors are mechanically active tissues, as it is hypothesized that biomimetic mechanical environments instruct immature cells to form differentiated tissues. One such tissue, skeletal muscle, has been identified as a key candidate for strain application due to the close structure-function relationship of myofibers. Here we detail the multiple uses of a custom-built bioreactor system in combination with electrospun fibrin microfibers for muscle tissue engineering. Outlined below are the methods used in the system to test the mechanical properties of hydrogel-based scaffolds in an aqueous environment, including Young’s modulus and poroelasticity. Additionally, we demonstrate the application of tensile strain to sterile cell cultures grown on electrospun scaffolds and perform end-point testing of tissue contractility with the addition of an electrode.

## Introduction

The field of skeletal muscle tissue engineering has long explored the role of tensile strain application as a driver of myogenic differentiation due to the mechanically active nature of skeletal muscle (Somers et al., 2017). Uniaxial tensile strain has also been shown to induce tissue alignment (Moon et al., 2008; Bayati et al., 2011; Pennisi et al., 2011; Egusa et al., 2013; Yilgor Huri et al., 2013; Heher et al., 2015), which in skeletal muscle is highly important, as myofibers must be aligned in the direction which they act to move or stabilize the bony skeleton. Early attempts to elucidate the role of strain in skeletal muscle differentiation began with 2D commercially available systems like the Flexcell^®^ tension system, on which cells can be uniaxially (Pennisi et al., 2011; Yilgor Huri et al., 2013; Dugan et al., 2014) or biaxially (Akimoto et al., 2001; Kumar et al., 2004; Kook et al., 2008a,b; Pennisi et al., 2011; Soltow et al., 2013) strained. However, studies have shown variability in the strain field of both biaxial and uniaxial Flexcell^®^ systems (Vande Geest et al., 2004; Colombo et al., 2008). This, in combination with the need to explore strain in 3D cultures, has led researchers to build custom bioreactor systems for skeletal muscle tissue engineering to improve usability of the system as well as allow for strain of 3D tissue constructs that more closely mimic the 3D *in vivo* environment (Powell et al., 2002; Moon et al., 2008; Heher et al., 2015; Cook et al., 2016; Li et al., 2016).

These 3D skeletal muscle bioreactors range from systems utilizing a stepper motor to apply strain (Powell et al., 2002; Moon et al., 2008; Cook et al., 2016) to systems that use magnetic elements (Heher et al., 2015; Li et al., 2016) to create strain. The differences in strain application also determine the variety of strain regimens that can be applied. With some magnetic systems, this means the system can only accommodate static strain (Li et al., 2016). Also of note is the number of constructs and types of constructs that can be used in the system. This can range greatly in size of construct, number of constructs and composition of constructs. Some systems are built around a four-well (Cook et al., 2016) or six-well plate (Powell et al., 2002), limiting the number of samples per run, while others are more flexible in their design, allowing for the strain of 10 (Moon et al., 2008) or even 36 (Heher et al., 2015) samples at once. Finally, the systems also vary in their ability to measure strains and adjust for pre-strain. Some systems lack a force-feedback mechanism, while others have force sensors that allow for the measurement of strain and force of contraction (Powell et al., 2002; Cook et al., 2016). In the present study, the designed bioreactor is among those with in-built force sensors, allowing us to utilize the system for several applications beyond that of strained culture.

Following the initial design and characterization of the bioreactor system (Cook et al., 2016), we have since used the system in many additional applications. The original intended use of the bioreactor to apply strains in a skeletal muscle tissue culture system has revealed that the maturity of myoblasts at the onset of strain application is an important factor in myogenic outcomes when static and cyclic strains are applied to maturing skeletal muscle grafts (Somers et al., 2019). Beyond this, the versatility of the system and the force sensors built into the bioreactor have allowed for the measurement of mechanical properties of electrospun fibrin microfibers (Zhang et al., 2014; Gilbert-Honick et al., 2020) and microfiber sheets of several different formulations to determine the appropriate formulation for matching the properties of native skeletal muscle (Gilbert-Honick et al., 2018) and cardiac muscle (Morrissette-McAlmon et al., 2020), respectively. More recently, we have also used the bioreactor to measure the stress relaxation of the electrospun fibrin microfibers and determine that the stress relaxation properties of the materials can be described with a poroelastic model (Yuan et al., 2018). Finally, we have also employed the bioreactor as a means to measure the force of contraction of matured muscle constructs, in both skeletal (Gilbert-Honick et al., 2018) and cardiac (Morrissette-McAlmon et al., 2020) tissue grafts. Detailed below are the protocols used with the bioreactor system for three applications: (1) measurement of material properties, (2) strained cell culture, and (3) force of contraction measurement.

## Materials and Equipment

### Bioreactor Parts and Assembly

i. Bioreactor housing
ii. Linear Stepper Motor (43H4C-2.33-815 Haydon Kerk Motion Solutions, Inc.)
iii. Motor face plate and screws
iv. Horizontal crossbar bar
v. Lower and upper walls and screws
vi. 4 linear actuators and pre-tensioning screws
vii. 4 linear actuator sleeves, clamps, and screws
viii. 4 force sensors (LQB630-50 G Cooper Instruments & Systems, Inc.) and attachment screws and spacers
ix. 4 force sensor sleeves, clamps, and screws
x. Electrospun samples
xi. Point electrode with platinum ends
xii. Power source for stimulation
xiii. Custom strain gauge amplification set up
xiv. Lab Jack U3-HV (LabJack Corp.)
xv. Programmable motor controller (PCM4826E Haydon Kerk Motion Solutions, Inc.)
xvi. Power source for motor controller
xvii. 2 pairs of sterile tweezers
xviii. 2 sterile Allen wrenches
xix. Sterile scissors
xx. Sterile Phillips head screwdriver
xxi. 2 sterile lab underpads
xxii. Sterile gloves
xxiii. Cell culture CO_2_ Incubator

#### Methods

**FIGURE 1:**
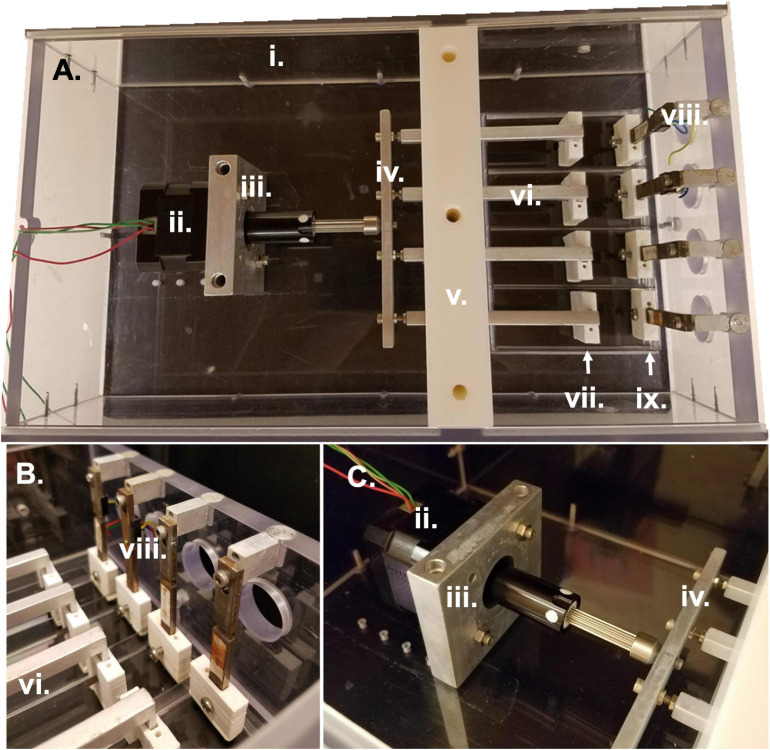
Bioreactor parts and assembly. **(A)** Overhead view of entire bioreactor system with actuation compartment to the left and sterile culture compartment to the right. **(B)** Side view of sterile culture compartment displaying the region between force sensors and linear actuators in which samples are clamped. **(C)** Side view of actuation compartment detailing the motor attachment to the linear actuators and bioreactor housing. All Roman numeral labels refer to the list of materials at the beginning of Materials and Equipment.

**FIGURE 2:**
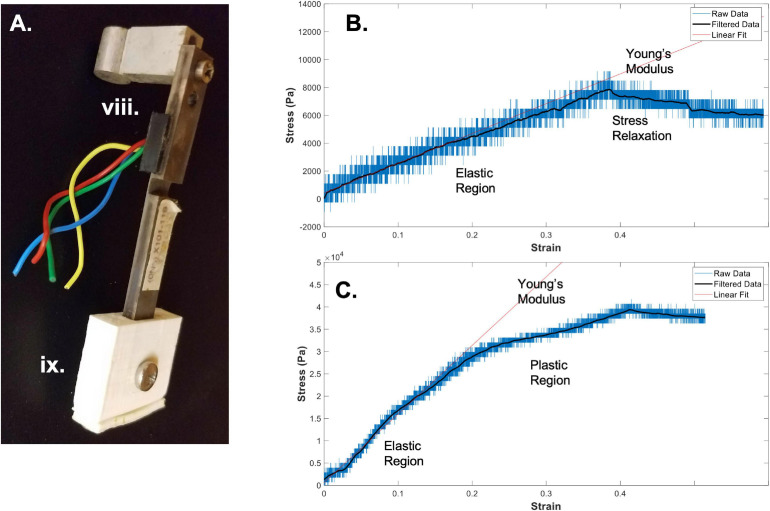
Bioreactor for mechanical testing. **(A)** Image of a force sensor, used as one end of the sample clamping, that allows for measurement of stresses during strain application to the sample. **(B)** A sample stress-strain curve from an electrospun fibrin microfiber measured with the bioreactor (Gilbert-Honick et al., 2020). **(C)** A sample stress-strain curve from an electrospun fibrin microfiber crosslinked with EDC/NHS chemistry as previously described (Gilbert-Honick et al., 2020). In both **(B,C)** strain was stopped at approximately 40% while measurement of force continued. Black line indicates filtered data. Roman numerals refer to the list of materials at the beginning of Materials and Equipment.

As previously described (Cook et al., 2016), the bioreactor system comprises a sterile culture compartment (Figure 1A right, Figure 1B) and an actuation compartment (Figure 1A left, Figure 1C). Within the sterile compartment are the force sensors and linear actuators, between which samples can be clamped. Both the force sensors and the linear actuators are protected by a custom-3D printed acrylonitrile butadiene styrene (ABS) plastic sleeve. The sleeves serve two purposes: (1) to prevent direct contact between the metal actuators or force sensors and the media to prevent contamination or rusting, and (2) to allow for clamping of the sample to each respective part of the system. The base of each sleeve has two screw holes allowing for the attachment of a small clamp piece (Figure 2A). This allows for the sample to be held between the base of the sleeve and the clamp piece using the two side screws. The custom-3D printed clamp piece also has topographical ridges printed on one side to improve contact between the sample and the clamp to reduce the risk of slipping. The force sensor and clamp-sleeve assembly serve as a post to which the sample is clamped. As previously described the signal from the force sensor is amplified by a universal strain gauge amplifier (Industrologic, Inc) and recorded by a LabJack U3-HV acquisition board. The optimal sensitivity of the system is about 50 μN (Cook et al., 2016).

The other side of the sample is clamped to the linear actuator and clamp-sleeve assembly. This end of the sample is moved via the programmable stepper motor which is mounted to the base of the bioreactor and attached to the four linear actuators through a horizontal connector bar (Figures 1A,C). The motor (43H4C-2.33-815, Haydon Kerk Motion Solutions, Inc.) has a step angle of 1.8° and microstepping capabilities (Full, 1/2, 1/4, 1/8, 1/16, 1/32, 1/64). The exact distance between the end of the linear actuator and the cross bar can be slightly modified to adjust for small differences in sample length or pre-tension upon loading in the bioreactor. The height of the four linear actuators is maintained by the center wall in the bioreactor which is composed of two pieces. The first piece is placed in the bioreactor and secured to the base. Following this, the linear actuators are placed into slots in the base piece of the wall and sandwiched in place by the top piece of the central wall. The wall pieces are made of a low friction material that allows the linear actuators to be simultaneously secured in their vertical position while still moveable in the horizontal plane to apply strain to the samples. The bioreactor also has two lid pieces that slide into the main housing of the bioreactor through grooves in the sides of the housing. These lids slot into a groove in the central wall to allow for a sterile environment.

### Application 1: Bioreactor for Measurement of Material Properties

One of the main applications of the bioreactor system is for the measurement of material properties. The system is particularly advantageous because it allows for measurement of materials in an aqueous environment, which is of particular interest to tissue engineers using hydrogel-based materials for *in vitro* and *in vivo* applications, both of which maintain the hydration of the sample. Many commonly used commercially available tensile mechanical testers do not allow the sample to maintain hydration throughout the testing period, especially because loading and clamping often wrings liquid out of the sample and because some tests, such as low strain rate modulus testing and stress relaxation testing, require long time periods of measurement during which the sample will dry. It is important to measure the sample in an environment that most closely mimics that in which it will be used, as the goal is to tune the material to mimic the native properties of the tissue which it will replace. It has been shown that matching the mechanical properties, such as stiffness (Engler et al., 2006) and viscoelasticity (Chaudhuri et al., 2015), of the material on which cells are grown to that of the native tissue toward which they are differentiating improves differentiation outcomes. For these reasons, we are particularly interested in using the bioreactor system to measure the material properties of scaffolds to allow us to tune the material to match native skeletal muscle in a hydrated environment.

Protocol for measurement of tensile material properties in a pre-assembled bioreactor:

1. Clamp the sample to be tested between the force sensor and the linear actuator.
  Prior to attempting to clamp the sample, make sure the distance between the linear actuator and the force sensor is appropriate for the length of the sample (We have found that ∼80% of the sample length between the ends of the two sleeves allows room for clamping). Clamping is most easily accomplished by inverting the bioreactor so that the base of the force sensor sleeve and the linear actuator sleeve are face up. Expediency can be quite important throughout the process, depending on how quickly the sample dries out when exposed to air. In the case of the electrospun fibrin microfibers, the samples dry quickly, especially once the samples are fully clamped, as the clamping process can wring out the fibers. For this reason, place a clamp onto each of the sleeves and begin securing the clamp by screwing one side of the clamp into the sleeve. Do not tighten fully so that you can swing the clamp out of the way to lay down your sample. For this system, because the microfiber is electrospun onto a frame, it is easiest to handle the sample while still on the frame and cut the frame away from the sample after clamping. This prevents damage to the sample from handling with tweezers and allows the sample to be placed directly perpendicular to the sleeves. If the sample is not placed directly perpendicular to the sleeve pieces, the tensile measurements will be inaccurate. Line up the frame and sample with the two sleeves (force sensor and linear actuator) and bring the clamp pieces back into position over the sample. Tighten the screws. If testing multiple samples at the same time, preparation for tightening the screws should be performed for all samples in an assembly-line fashion before tightening the screws as the samples dry out quickly once the screws are tightened. Once the screws are tightened, the sides of the frame that would prevent the sample from being extended should be cut and the bioreactor assembly should be flipped back to the upright position over a four-well plate pre-filled with water or another media to keep the samples hydrated during testing.
2. Extend the motor to create slack length in the sample(s).
  Following clamping of the samples into the bioreactor assembly, measurements of tensile moduli can be improved by extending the motor so that the sample begins in a slack position. This is to eliminate any potential pre-tension in the samples upon loading. Depending on the sample type and mode of clamping into the system, this step may or may not be necessary. The motor can be extended by using the RealTime tab in the IDEA Drive (Haydon Kerk) software or can be controlled in a similar way by another motor system. Again, depending on the sample and loading conditions, the amount of extension necessary to remove any pre-strain could vary. An extension of 10% of the original length of the sample has worked well for our experiments.
3. Retract the motor to begin straining the sample.
  Once the sample is clamped and slightly slack, straining can begin. When choosing a strain regimen to measure the tensile Young’s modulus, keep in mind that the sample is beginning in a slack position, meaning that to achieve a 50% strain you may have to extended it beyond 50% of the original length of the sample to make up for the initial slack length. The strain rate and distance can again be controlled through the RealTime tab of the IDEA Drive software or with a similar motor system-controller setup. Additionally, to perform the strain without the motor connected to a computer, pre-program a set of commands and download it to the motor controller. Before beginning the strain regimen, ensure that the values of the force sensors of interest are being written and recorded in the Lab Jack software to which they are connected. Also ensure that the frequency of data collection of the force sensors is much greater than the step rate to ensure acquisition of sufficient data. The strain regimen can be tailored to any specific experimental question. An example of data acquired using a strain rate of ∼1%/ sec with a fibrin fiber sample strained to ∼40% beyond its original length can be seen in Figure 2B (Gilbert-Honick et al., 2020). Modification of this type of sample using N-Ethyl-N’-(3-dimethylaminopropyl) carbodiimide hydrochloride (EDC) and N-hydroxysuccinimide (NHS), referred to as EDC/NHS crosslinking chemistry, both applied to the electrospun sample at 10 mg/ml, significantly modifies its mechanical properties (Figure 2C; Gilbert-Honick et al., 2020). In previous work, we have tested strain rates ranging from 0.25%/s to 3%/s (Yuan et al., 2018). We have also used microstepping in many of these tests (1/8 or less down to 1/64) to reduce noise from the motor. Tests were performed with the default acceleration of zero which indicates that the extension began at the specified run speed.
4. To measure stress relaxation, hold the sample at strain length following strain application.
  In addition to measuring the Young’s modulus of materials using the bioreactor system, we have also used the bioreactor system to measure the stress-relaxation properties of the fibrin microfibers (Yuan et al., 2018). This can be accomplished by continuing to measure output from the force sensor following the extension of the fibrin microfiber. An example of the type of relaxation seen in the fibrin microfiber material can be seen following the cessation of strain in Figure 2B. Similarly, hysteresis curves could be acquired in the system with the proper strain regimen.
5. Conversion of voltage measurements from the force sensor to a stress-strain curve for measurement of Young’s modulus.
  In order to convert the measurements from the force sensor to stress-strain curves, the change in voltage must first be converted to a change in force. The voltage-force relationship of the force sensors can be determined by applying a known mass to the end of the bending beam with the beam parallel to the ground. Due to the sensitive nature of the force sensors used with the system, we have found that droplets of water of known volume (and therefore known mass) are the most reliable method for achieving a force-voltage plot. The slope of this plot acts as a conversion factor from voltage to force. Furthermore, force can be converted to stress by dividing by the cross-sectional area of the fiber. In the case of the fibrin microfibers, we measure the diameter of the fiber using brightfield images and estimate the cross-sectional area as a perfect circle. The strain corresponding to the measured stress can be determined by converting the time output to strain using the strain rate. The strain can be zeroed and the initial length of the fiber adjusted based on the time at which the force reading departs from the baseline (i.e., when the fiber is no longer slack). Due to the sensitivity of the force sensors, the data can often be noisy from the rotation of the stepper motor and other external movements. This can often be cleaned using a Savitzky-Golay filter (Savitzky and Golay, 1964), as indicated by filtered data (black line) in Figures 2B,C. In this circumstance a filter with an order of 1 and frame length of 391 was used based on the number of data points present within each noise cycle. Young’s modulus of the material can be determined by fitting a linear line to the adjusted data in the linear elastic region.

### Application 2: Bioreactor for Strained Cell Culture

Although the bioreactor system works well for measurement of mechanical properties, the original purpose for which it was built was strain application to cell constructs in a sterile condition. This is of particular interest for use in the field of tissue engineering, in which it has been shown that biomimetic mechanical loading can aid in differentiation outcomes for multiple tissue types (Mauck et al., 2000; Donahue et al., 2001; Akhyari et al., 2002; Altman et al., 2002; Powell et al., 2002; Hung et al., 2004; Moon et al., 2008; Heher et al., 2015; Li et al., 2015). We have used the bioreactor system for the application of strain to skeletal muscle progenitors (C2C12 mouse myoblasts, ATCC) (Somers et al., 2019). However, the system could be used for several different tissue types and scaffold-cell combinations. The bioreactor is highly versatile, allowing for up to four constructs to be loaded at different starting lengths and tensions. Additionally, the bioreactor can accommodate tissues from 20 to 60 mm with an adjustable motor arm length and positioning. The programmable motor also allows for the application of hundreds of different strain regimens due to its small step size and highly adjustable programmable functions (acceleration, speed, wait time between steps, etc.). All of these advantages make the system uniquely adaptable to the challenges of engineering skeletal muscle and other mechanically active tissues.

**FIGURE 3:**
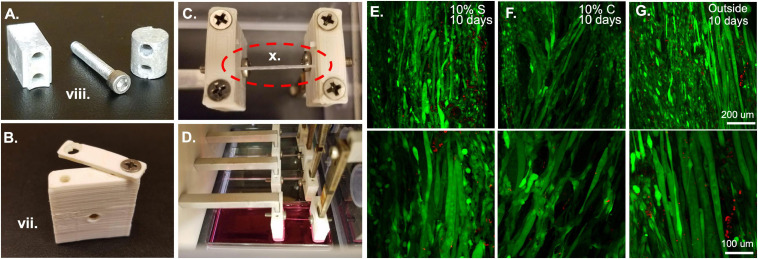
Bioreactor for strain application. **(A)** Metal components of the bioreactor, such as these force sensor attachment screws and spacers are autoclaved prior to sterile assembly. **(B)** Plastic ABS pieces such as this sleeve and clamp are soaked overnight in 70% ethanol prior to bioreactor assembly. **(C)** Following assembly of the system, fibrin microfiber samples are clamped into place in the inverted bioreactor. **(D)** Once the fibers are clamped, the bioreactor is flipped upright, and the sample is placed in media for culture. **(E)** Initial tests of 10% static and **(F)** 10% cyclic strain in the bioreactor demonstrate cell survival through live (green)/dead (red) staining similar to that of **(G)** cells grown outside the bioreactor. Roman numerals refer to the list of materials at the beginning of Materials and Equipment.

Protocol for sterile assembly of the bioreactor system and strain application to C2C12 and fibrin microfiber skeletal muscle constructs in the bioreactor:

1. Prepare all pieces for assembly.
  In the bioreactor system, all metal pieces of the bioreactor are autoclaved. This includes all the screws for the clamp assemblies, the wall screws, the motor face plate and screws, and the screws and spacers for the force sensor attachment to the bioreactor housing (Figure 3A). In addition to these pieces, autoclave two sets of tweezers, two Allen wrenches, a Phillips head screwdriver, and two lab underpads. All ABS pieces that are in contact with the cells and media should be soaked overnight in 70% ethanol (Figure 3B). The plastic bioreactor housing can be vigorously sprayed down with 70% ethanol on the day of assembly to avoid cracking the plastic by leaving it in 70% ethanol overnight. The force sensors should be soaked in 70% ethanol up to the wire region for at least 30 min before assembly and the top of the force sensors (wire region and above) should be sprayed with 70% ethanol carefully to sterilize without submerging the wires.

2. Assemble clamps.
  Assembly of the bioreactor system requires two people. One person should wear regular gloves (person A), while the other, who will handle all components to be used in the sterile compartment, should wear sterile, surgical gloves (person B). Person A should handle all movement of materials in and out of the sterile hood and open all autoclaved packages to ensure that person B can maintain the sterility of their gloves.
  Before beginning any assembly, the two autoclaved lab underpads should be opened in the hood and laid out, one surface for person A, on which all 70% ethanol sprayed objects and autoclaved pouches will be placed, and one for person B, on which only sterile elements and autoclaved pieces will be placed. The first task, which requires the most care to ensure a sterile field, is putting together the clamp and sleeve assemblies which will be in contact with the samples and the media. To do this, person A should open the autoclaved packet of tools and place them in a mutually accessible position. One set of tweezers and one Allen wrench should be reserved for each person. Since person A is not wearing sterile gloves, tools should not be shared. Person A should empty the package of autoclaved clamp screws onto person B’s underpad. To ensure that the samples do not get exposed to 70% ethanol, person A should begin by washing all the ABS clamp and sleeve pieces. This can be accomplished by moving the pieces from the beaker of 70% ethanol to a beaker of sterile water with tweezers. The pieces then should be moved to a third beaker, again containing fresh sterile water, for a second wash. Following the second wash they can be moved to the sterile underpad in front of person B. Person B can begin assembling the clamp and cover combinations by screwing a screw into one side of the clamp and sleeve. This should be done with the sterile gloves or the sterile Phillips head screwdriver. The screw should not be made too tight so that the clamp is still rotatable for the sample clamping procedure (Figure 3B). Repeat this until there are four clamp-force sleeve assemblies and four clamp-linear actuator sleeve assemblies.
3. Prepare the Motor and linear actuators.
  During autoclaving, all pieces of the linear actuators (four L-pieces, horizontal crossbar, and pre-tensioning screws, which are all metal) may be kept together and autoclaved as one piece. This makes for a speedier assembly process. Person A should open the autoclave pouch with the linear actuators and empty the piece onto the sterile underpad of person B. At this time, person B should slide the linear actuator sleeves (with clamps attached) onto the ends of the linear actuators. Each sleeve has a hole that aligns with a hole in the end of each linear actuator that a sterile screw should be put through to keep the covers from sliding off the ends of the linear actuators. While person B is completing the assembly of the sterile region of the linear actuators, person A can prepare the motor to attach to the linear actuators.
  The motor should be sprayed with 70% ethanol to avoid contamination, but care should be taken to avoid wetting the wires. The motor face plate, which can be autoclaved with its screws, should be attached to the motor. The face plate has two vertical canals through which screws can be used to attach the motor assembly to the base of the bioreactor housing (Figures 1A,C).
  Once person B has completed covering the ends of the linear actuator with sleeves, they should mount the linear actuators onto the end of the motor, which is threaded to match the threads of the horizontal crossbar connecting all of the linear actuators. Person B should avoid touching the motor during this step to maintain the sterility of their gloves. If necessary, person A can help stabilize the motor during the attachment process. At this time, the motor-linear actuator assembly is ready to be placed in the bioreactor housing. The bioreactor housing, including the lower wall, can be pre-assembled and should be sprayed down with 70% ethanol vigorously, wiped, and sprayed again before bringing it into the hood. A sacrificial four-well plate should be put into the plate space temporarily to avoid the clamp regions from contacting the hood surface. The four linear actuators slot into the grooves of the lower wall. Align the motor face plate with one of the sets of the holes at the back of the non-sterile compartment. There are multiple hole options to allow for samples of different dimensions. Person A should then screw the motor face plate to the base of the bioreactor using one of the Allen wrenches. Following this, they can also screw the top portion of the center wall on to secure the linear actuators in their grooves.
4. Place force sensors into bioreactor housing.
  Meanwhile, person B can begin slotting the other clamp-sleeve assemblies onto the ends of the force sensors. This step should be the last time person B considers their gloves sterile because the top region of the force sensors has only been sprayed with 70% ethanol. Therefore, one hand can be used to stabilize the force sensor while the other is used to touch the sterile sleeve-clamp assembly, or the sleeve-clamp assembly can be placed onto the end of the force sensors with sterile tweezers. Again, a sterile screw should be put through the hole in the middle of the sleeve and force sensor to secure the vertical position of the sleeve on the force sensor. Following this step, the metal spacers and screws seen in Figure 3A should be attached to the top of the force sensor. The cylindrical portion is attached to the force sensor with the rectangular piece and screw. The cylindrical portion then slots directly into the wall of the bioreactor (Figures 1A,C). Avoid putting too much torque on the force sensors during this step, as they are highly sensitive and could be broken by too much force. This should be done with a four-well plate still in the base of the bioreactor to avoid any contact with the hood. At this stage constructs can be placed in the bioreactor for straining. Note: if assembling multiple bioreactors, or to save time on the first day of cell straining, this is a good stopping point. Leave the bioreactor in the sterile hood and ensure that the lids of the sterile and actuator compartments are on and any holes for wires are sealed.
5. Clamp electrospun constructs into clamps.
  After the completion of the bioreactor assembly, measure the distance between the linear actuator clamps and the force sensor clamps to make sure the distance is appropriate for the sample length, like the procedure in Application 1.1. Again, timing is key during the clamping process to ensure that the samples do not dry out. Samples can be clamped in several different manners, for example, an acellular sample can be clamped into the bioreactor and then seeded with cells (Figure 3C). Or a pre-seeded construct can be placed in the bioreactor for strain. If the samples are pre-seeded, make sure the cells have had an appropriate amount of time to attach to the construct before placing them into the bioreactor system. The samples are clamped by keeping them on the frame for handling purposes, placing them over the clamp regions with one screw already in place, and then bringing the clamp piece around from an open position (Figure 3B) to a closed position. This procedure should be done for all samples in each bioreactor before the second screw is put in place and tightened. This improves the hydration of the samples. Also, if cells are already seeded on the scaffold, have a second person assist in dripping media onto the samples during this process to maintain hydration of the cells. At this point, adjust the pre-strain screws if the samples are slack. Since the four samples are all strained by the same motor, all samples must be of a similar length to allow for the same strain regimen application. This would also be an appropriate time to seed cells onto acellular scaffolds. To accomplish this, concentrate the cells in a small volume of media (∼15–40 μl) and drip them directly onto the construct. If the cells are seeded in an excess of media, droplets will form and drip off the construct, making seeding efficiency low and inconsistent between samples. Following construct clamping, cut the edges of the frame from between the clamps so that only the samples remain (Figure 3C) and flip the bioreactor into place over a four-well plate filled with media (Figure 3D).
6. Application of strain to tissue constructs.
  Once the constructs are seeded, clamped, and submerged in media, again ensure that all wiring and screw holes are sealed before placing the bioreactor into the incubator. At this point, a straining regimen on the tissue constructs can begin. If the cells were only recently seeded, consider observing a waiting period to allow the cells to attach and adjust to their new environment. Strain is applied through the programmed movement of the stepper motor. This is achieved by programming a motor control unit. When the motor control unit is powered and connected to a computer, using the IDEA drive software, use the programming tab to add repeated extensions and retractions of the motor to create a strain regimen. The speed of extension and retraction determine the frequency of strain in a cyclic strain regimen and wait times after or before strain can be used to create a custom static strain regimen. After programming the strain regimen, download the program to the motor controller and select the saved program as the start-up program. This means that as soon as the motor controller is powered up and connected to a motor the program will begin allowing strain application to your constructs without having the motor controller plugged into a computer. Plug the motor controller into the motor in the bioreactor and place the motor controller on top of the bioreactor, then plug the motor controller into a power source (kept outside the incubator). As the motor controller receives power, the strain regimen will begin. A delay can be included at the beginning of the regimen if the moving parts constitute a safety concern. The motor controller can also be used while still connected to a computer if this is deemed more appropriate. Live dead staining of samples strained statically (Figure 3E) and cyclically (Figure 3F) in the bioreactor system show comparable amounts of live cells to those outside of the bioreactor (Figure 3G) demonstrating the ability of the bioreactor system to maintain cell viability. In the images shown here we have tested 10% strain applied statically or cyclically over 6 h with 3% strain in between. Cyclic strain was applied in a triangular wave at 0.5 Hz frequency, oscillating between 10 and 3% strain. Acceleration was left at the default, 0, which indicates that the extension began at the specified run speed.

### Application 3: Bioreactor for Force of Contraction Measurement

**FIGURE 4:**
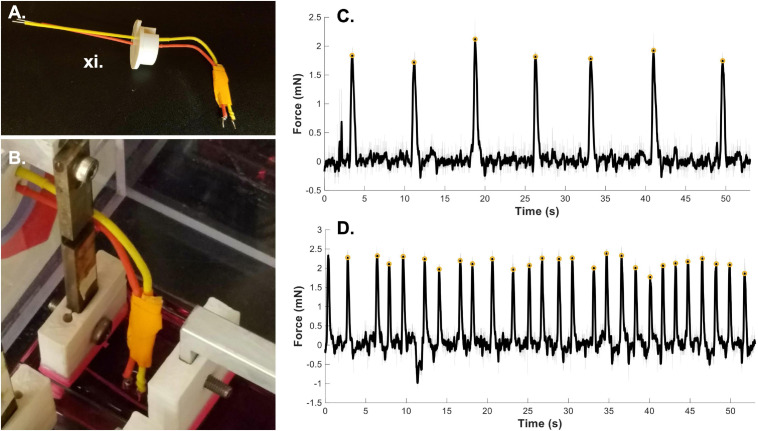
Bioreactor for force of contraction measurement. **(A)** Stimulated force of contraction is achieved through the use of a point electrode. **(B)** The electrode is placed in the media bath directly next to the sample. A fibrin microfiber used for skeletal muscle constructs is shown here next to the electrode. **(C)** A sample graph of spontaneous contractions of a cardiac tissue graft (Morrissette-McAlmon et al., 2020) demonstrates the ability of the bioreactor to measure spontaneous contractions. **(D)** A sample graph of stimulated force of contraction of a cardiac tissue graft measured by the bioreactor system (Morrissette-McAlmon et al., 2020). Gray lines indicate raw data, black lines indicate smoothed data, and yellow circles indicate measured peak forces. Roman numerals refer to the list of materials at the beginning of Materials and Equipment.

In addition to the ability to culture constructs in the bioreactor, the system has the advantage of also allowing for post-culture measurement of the contractility of tissues. This is particularly advantageous for tissues that function as contractile units: we have performed endpoint testing on skeletal and cardiac muscle grafts using the bioreactor system to measure their functional maturation (Gilbert-Honick et al., 2018; Morrissette-McAlmon et al., 2020). Because one of the clamping ends of each construct is attached to a force sensor, the system, with the addition of an electrode (Figures 4A,B), lends itself to measuring force of spontaneous (Figure 4C) and stimulated (Figure 4D) contractions. Thus far, we have used the bioreactor as an endpoint measurement following the growth and maturation of tissue grafts; however, the bioreactors can potentially be used to measure contraction throughout the time course of differentiation as well.

1. Clamp sample into the bioreactor system.
  Begin with a fully assembled bioreactor system. If the system is only being used to provide an endpoint test, the bioreactor can be assembled in a non-sterile environment. As in Application 1.1 and 2.5, ensure that the distance between the two clamp ends is appropriate for the length of your sample. Adjust, as necessary. Clamp the sample between the two clamp ends, working with haste so that the live sample does not dry out. Since the samples are on frames (both skeletal muscle fibers and cardiac muscle sheets), we can install them at an appropriate resting length. Cut the frame from the sides of the sample to allow for contraction against the clamp ends and flip the sample into pre-warmed media. If the sample is slack, the motor controller and/or pre-tensioning screws can be used to adjust the sample length, however, take care not to damage the tissue construct by straining it too far past its resting length. To optimize measurements, the tissue construct should be kept warm during testing. If possible, place the bioreactor in a non-sterile incubator. If you do not have an incubator for this use, place the bioreactor on a heating pad to keep the media warm.
2. Measure spontaneous contractions.
  To measure spontaneous contractions (Figure 4C), allow the sample to adjust to the new media and come to temperature for a few minutes. Then plug the force sensor lead wires into a custom Lab Jack based readout system (Cook et al., 2016). Begin recording the output to the Lab Jack software. If spontaneous contractions are present, you will see changes in the voltage corresponding to forceful contractions. This output will allow you to measure the force of spontaneous contractions as well as the frequency of contraction.
3. Position electrode and measure forced contractions.
  To measure stimulated force of contraction, an electrode must be placed in the well with the sample. The point electrode consists of a positive and negative lead (Figure 4A, red and yellow) with a platinum end that contacts the media. Place the electrode in media close to the sample without making contact (Figure 4B). For the bioreactor system we have 3-D printed an electrode holder that fits into the bioreactor wall where the force sensor wires exit. This allows for stabilization of the electrode position. The leads of the electrode can be connected to a stimulation source, either custom built or commercially available. In the case of stimulation of cardiac muscle grafts (Figure 4D), samples were stimulated at 0.5 Hz with a 10V DC, 20 ms pulse. This can be adjusted based on the sample type, stimulation source and desired question to be answered. For example, a longer AC pulse could be used to measure tetanic contractions. The sample length can also be adjusted using the motor and tested again to develop a force-length relationship plot. Since the force sensors are also voltage based, it is imperative that the force sensors have no direct contact with the media and therefore the stimulation voltage. Keep this in mind when designing force sleeve covers and when installing hydrated samples into the bioreactor. In previous work, we have tested the force-length relationship of cardiac constructs by straining the sample in 2% increments up to 20% strain and measuring force of contraction at each length (Morrissette-McAlmon et al., 2020). Raw voltage data can be converted to force data using the same methodology as described in application 1.5.

## Anticipated Results

Using the methods outlined above, we have successfully used the bioreactor for all three detailed applications: hydrated mechanical testing, strained sterile culture, and force of contraction measurement. When measuring the mechanical properties of electrospun fibrin microfibers for skeletal muscle applications, we can achieve stress-strain graphs such as those shown in Figure 2B, in which there is a clear linear elastic response to the strain application, as well as a visible stress relaxation following the time at which the motor was stopped (Gilbert-Honick et al., 2020). We have also used the bioreactor to further probe the stress relaxation response of materials as a function of poroelasticity (Yuan et al., 2018). Additionally, we have utilized the bioreactor system to compare the mechanical properties of control electrospun fibrin microfibers to those treated for (EDC/NHS) cross-linking, a method we have previously used to tether agrin to fibrin constructs to encourage neuro-muscular junction improvement (Gilbert-Honick et al., 2020). A sample stress-strain graph of the mechanical properties of the treated fibrin microfibers (Figure 2C) shows that the cross-linking increases the tensile Young’s modulus of the material and is not linear beyond ∼20% strain, indicating a potential plastic response beyond this amount of strain (Gilbert-Honick et al., 2020). In both of these cases, the noise of the system, likely caused by motor vibrations, can be greatly reduced through the application of a Savitzky-Golay filter (Figures 2B,C black line). These data demonstrate the use of the bioreactor as a mechanical tester that answers questions regarding the material properties of hydrogel-based systems we would be unable to answer with dry, open-air testing.

When straining cell-laden constructs in the bioreactor, we have had great success with cell survival (Figures 3E–G) indicating that the bioreactor and pieces are suitable for sterile cell growth and the cells we utilize (C2C12s) can survive the strain regimens. We tested constructs held at 0% strain for 3 days followed by static (Figure 3E) and cyclic (Figure 3F) 10% strain for 6 h a day for 7 days. While not under strain or at the lowest point of the cyclic strain cycles, the samples were maintained at 3% strain based on the work of Heher et al. (2015). Following 10 days, the samples showed comparable survival to unstrained samples outside of the bioreactor (Figure 3G). These results demonstrate the survival of cells in the bioreactor. We have also successfully demonstrated the role of strain timing in changing myogenic outcomes using the bioreactor system (Somers et al., 2019). In this case, the samples were pre-grown outside of the bioreactor and placed in the bioreactor after 3 or 5 days of growth, indicating that seeding the samples in the bioreactor or before installation in the bioreactor are both viable methods for straining cell constructs.

Finally, we have demonstrated the use of the bioreactor system for end-point testing of force of contraction. As previously shown, we have successfully measured the spontaneous (Figure 4C) and stimulated (Figure 4D) force of contraction of cardiac muscle graphs on electrospun fibrin sheets (Morrissette-McAlmon et al., 2020). The “smooth” function in MATLAB was utilized to remove noise from the signal (filtered black line) and a custom MATLAB script using the “find peaks” function was used to find the amplitude of the force of contraction (yellow circles). The same MATLAB script was also used to determine the average frequency of spontaneous contraction and determine and correct for any fluctuations in the baseline voltage reading. This use of the bioreactor allows for testing of the functional maturity of a variety of constructs and allows for several different measurements including spontaneous force of contraction, stimulated force of contraction, and observation of force-length relationships.

## Discussion

In this work, we demonstrate several novel uses of the bioreactor system with tissue engineering applications. In addition to its main intended use as a strain system for sterile tissue culture, the system can also be used for hydrated mechanical testing of tissue engineered constructs. This allows for exploration of tensile mechanical properties of hydrogel-based scaffolds that would be greatly different if tested in a traditional, dry environment. We have also outlined the use of the bioreactor system as an end-point test of force of contraction, allowing us to measure functional outcomes of engineered tissues.

In addition to the work demonstrated here, future improvements to the system could make it even more versatile. For the improvement of hydrated mechanical testing methods, we could purchase force sensors of lower and higher maximum limits to test materials considerably softer or stiffer than the ones we currently use. Also, replacing the stepper motor with a servomotor would help reduce the noise in the stress-strain curves caused by the vibration of motor stepping. Including an optical encoder or laser-based sensor in tandem with the current setup could also improve stress-strain curves in giving direct positional data instead of relying on calculated position based on the strain rate.

The most challenging aspect of strained sterile culture in the bioreactor system is the mechanism for clamping the samples, especially when samples are already seeded with cells. The expediency necessary to maintain cell survival and construct hydration during this period could be daunting for a new user of the system. Therefore, in the future, the use of a spring-loaded or magnetized clamp could be more advantageous than the use of a screw-based clamp to reduce the amount of time the samples are exposed to the air. This is one advantage of other systems in the field that use magnetized samples (Li et al., 2016) or a spool and hook system with a circular construct (Heher et al., 2015). Additionally, the clamps could be improved by changing the material from which they are printed. ABS printing causes innate porosity that could eventually lead to contamination issues if the pieces are not changed out on a normal basis. The material could be switched to a less porous, autoclavable, and printable material such as carbon-fiber reinforced nylon (Blok et al., 2018) or could be machined out of another plastic such as Teflon.

The one-motor bioreactor system has many advantages, making it high-throughput and affordable. However, the single motor design is limiting in that all four samples must receive the same strain regimen and if there are considerable differences in the length or pre-straining conditions of the samples, the strain percentage applied between different samples will slightly vary. This could be improved if multiple motors were included in each bioreactor to control different samples. For our purposes, however, the one motor design has worked well, as we are using four of the same scaffolds and have multiple of the same bioreactor to allow for comparison of different regimens.

For measurements of contraction, the current system could be improved by the inclusion of more sensitive force sensors to measure contractions that are not currently measurable with the existing force sensors, or not distinguishable from the noise of the system. Also, because of the voltage-based nature of the force sensors, if the force sensor comes into contact with any liquid, the stimulating voltage can cause an artifact in the force sensor readout. This is mostly an issue with broader pulse stimuli where the pulse could be mistaken for a contraction. To ensure this is not happening, following testing, the sample can be cut from one clamp end and the system can be tested again. If the same signal is present on the force sensor readout, the force sensor is reading the stimulus and the signal cannot be from contraction. To avoid problems with this in the future, a different type of force sensor (i.e., non-voltage based) could be used, or a different method of functional testing could be employed. For example, in the field of skeletal muscle tissue engineering both Ca^2+^ transient (Hinds et al., 2011; Juhas et al., 2014; Madden et al., 2015; Rao et al., 2018) and optical force transducer methods (Hinds et al., 2011; Juhas et al., 2014; Madden et al., 2015; Rao et al., 2018) have been utilized outside of bioreactor systems to test tissue functionality. Additionally, a hydrophobic coating or less porous sleeve could be placed on the current force sensors to ensure no liquid is leaking through the force sensor sleeve and interfering with the voltage readings.

## Conclusion

In the future, this system may be used for additional applications. For example, the mechanical testing application could be further extended to test not only the acellular scaffolds, but the developing tissue construct over time to determine how the cells are modifying the mechanics of the tissue construct. Also, other cell and tissue types could be strained in the bioreactor to study the role of strain in other mechanically active tissue types like cardiac muscle, tendon, and ligament. In the future the force of contraction measurement function of the bioreactor will be employed to measure contractions of tissues throughout growth. The system could also be modified to deliver electrical stimulation in a sterile manner throughout the growth and differentiation phase to measure stimulated force of contraction or as an additional maturation cue. Measurement of stimulated force of contraction could also be adapted to include a field electrode instead of the current point electrode in use. Nevertheless, the bioreactor system in its current state has been an invaluable tool for mechanical testing, sterile culture, and functional testing applications in the lab.

## Data Availability Statement

The original contributions presented in the study are included in the article/supplementary material, further inquiries can be directed to the corresponding author/s.

## Author Contributions

SS and WG wrote the manuscript. Both authors contributed to the article and approved the submitted version.

## Conflict of Interest

The authors declare that the research was conducted in the absence of any commercial or financial relationships that could be construed as a potential conflict of interest.
